# An Appraisal of the Role of the Neocerebellum for Spatial Navigation in Healthy Aging

**DOI:** 10.1007/s12311-022-01389-1

**Published:** 2022-03-08

**Authors:** Stephen Ramanoël, Marion Durteste, Victor Perot, Christophe Habas, Angelo Arleo

**Affiliations:** 1 Sorbonne Université, INSERM, CNRS, Institut de la Vision, 17 rue Moreau, F-75012 Paris, France; 2 Université Côte d’Azur, LAMHESS, Nice, France; 3 CHNO Des Quinze-Vingts, INSERM-DGOS CIC 1423, 28 rue de Charenton, 75012 Paris, France; 4 Université Versailles St Quentin en Yvelines, Paris Saclay, 78180 Montigny-Le-Bretonneux, France

**Keywords:** Navigation, Cerebellum, Healthy aging, MRI, VBM

## Abstract

Spatial navigation is an intricate ability, requiring multisensory and motor integration, that is particularly impacted in aging. The age-related decline in navigational capabilities is known to be associated with changes in brain regions such as the frontal, temporal, and cerebellar cortices. Age-related cerebellar differences in spatial navigation have generally been ascribed to motor impairments, omitting the central role of this structure in several cognitive processes. In the present voxel-based morphometric study, we investigated gray matter volume loss in older adults across cognitive and motor subregions of the cerebellum. Specifically, we hypothesized that age-related gray matter differences would occur mainly in cerebellar regions involved in cognitive processing. Our results showed a significant age-related atrophy in the left neocerebellum of healthy older adults that includes Crus I and lobule VI. The latter are important nodes in the network that subtends cognitive abilities such as object recognition and spatial cognition. This exploratory work sets the ground for future research to investigate the extent of the neocerebellum’s contribution to spatial navigation deficits in aging.

## Introduction

Navigating in space constitutes a key factor to maintain older adults’ autonomy and mobility and to prevent the occurrence of age-related disorders. Healthy older adults show impairments in spatial navigation ability that include disorientation in unfamiliar environments and difficulties in taking detours to avoid obstacles [1]. This decline in navigational capabilities is associated with anatomical and functional changes across numerous interconnected structures of the brain including the cerebellum [1, 2]. Accumulating evidence indicates that cerebellar functioning extends beyond the scope of motor control and that multiple cerebellar subregions play a central role in various aspects of cognition such as attentional, executive, social and emotional processing [3–8]. Indeed, the neocerebellum takes part in various interconnected circuits including the central executive, default mode, and dorsal attentional networks that subserve general cognitive and affective functions [6]. The cerebellar cortex appears to be organized into three functional gradients that code for increasingly abstract processes [7]. In addition, motor and cognitive cerebellar territories are thought to compute contextual forward models that enable precise timing, sequencing, optimization, and automation of mental processes [8]. Recently, King and colleagues developed a task-related functional atlas of the cerebellum evidencing distinctions between regions involved in motor or cognitive processing [4]. Strengthening the role of the cerebellum in spatial cognition, two recent meta-analyses of neuroimaging studies on spatial cognition [9, 10] showed that cerebellar regions in interaction with frontal, parietal, and temporal cortical regions were related to the non-motor aspects of spatial navigation tasks. In the same vein, a functional magnetic resonance imaging (fMRI) study found that a hippocampo-cerebellar network mediates the use of spatial strategies during navigation [11]. Notably, Igloi and colleagues reported that right Crus I in relation with medial prefrontal cortex and left hippocampus contributes to sequence-based navigation, whereas left Crus I in conjunction with right hippocampus and medial parietal cortex underlies place-based navigation.

In the healthy aging literature, a handful of studies have reported age-related changes in cerebellar volume or activity associated with worse navigational performance [2]. However, these studies often failed to provide any interpretation of results pertaining to the cerebellum and, if they did, explained such age-related differences in terms of declining voluntary motor control. Hence, the contribution of cognitive subregions of the cerebellum to the decline of navigational abilities in healthy aging deserves greater attention. We analyzed morphological data from a previous fMRI study in which young and older participants performed a virtual spatial navigation task [12]. This study revealed a decrease in navigational performance in older adults, associated with vast changes in neural activity in the cortical network subtending spatial cognition. In the present exploratory study, we looked at gray matter (GM) volume differences between young and healthy older adults in the cerebellum. First, we examined whether age-related differences in GM volume would be localized in cerebellar regions underlying either motor or cognitive functions. Second, we studied the associations between behavioral performance and cerebellar GM volume.

## Materials and Methods

### Participants and Task

The present study is a re-analysis of anatomical data from 25 younger (age 22–32 years old; mean 25.4 years; 7 females and 15 males) and 17 older participants (age 67–81 years old; mean 73.0 years; 10 females and 7 males). All participants completed a battery of neuropsychological tests including the Mini-Mental State Examination (MMSE; [13]), the 3D mental rotation test (3D; [14]), the perspective taking test (PPT; [15]), and the Corsi block-tapping task [16]. Inside the MRI scanner, subjects were asked to remember the position of a hidden goal in a three-armed maze and navigate back to it from different starting positions. To this end, they had to make use of three distinct 3D objects (a square, a triangle, and a sphere) placed in the center of the maze. The navigation time (time to reach the goal) and the error rate (proportion of wrong turns across trials) were recorded. Following the MRI experiment, participants completed an informal questionnaire to assess their preference for response-based or place-based navigation strategies. Additional participant and task information can be found in [12]. All subjects provided written informed consent, and the study was approved by the Ethical Committee “CPP Ile de France V” (ID_RCB 2015-A01094-45, CPP N°: 16,122).

### MRI Acquisition

Data were acquired using a 3-Tesla Siemens MAGNETOM Skyra whole-body MRI scanner (Siemens Medical Solutions, Erlangen, Germany) equipped with a 64-channel head coil. T1-weighted high-resolution three-dimensional images were obtained using an MPRAGE sequence (voxel size = 1 × 1 × 1.2 mm, TR/TE/IT/flip angle = 2300 ms/2.9 ms/900 ms/9°, matrix size = 256 × 240 × 176).

### MRI Analyses

Data processing was performed using the cerebellar-specific toolbox SUIT [17–19] and the general linear model [20] with SPM12 (Wellcome Department of Imaging Neuroscience, London, UK, http://www.fil.ion.ucl.ac.uk/spm/) implemented in MATLAB 9.4. Each individual scan was manually reoriented to set the origin (0, 0, 0) on the anterior commissure and thus isolate segmented maps of the cerebellum. Manual quality control was performed to correct potential segmentation errors such as GM located outside of the cerebellum. Images were then normalized using the diffeomorphic anatomical registration through exponentiated lie algebra algorithm (DARTEL) [21, 22], modulated and smoothed using a 6-mm FWHM Gaussian Kernel. We first performed a two-samples *t*-test to compare GM volume between young and older adults. Second, we performed a multiple regression analysis to investigate the associations between navigation time and error rate and GM volume of cerebellar subregions. For all analyses, brain size and gender were included as covariates of no interest. The statistical threshold was set at *p* < 0.05 family-wise-error (FWE) corrected at cluster-level with a minimum cluster extent of 20 voxels.

## Results

**Fig. 1:**
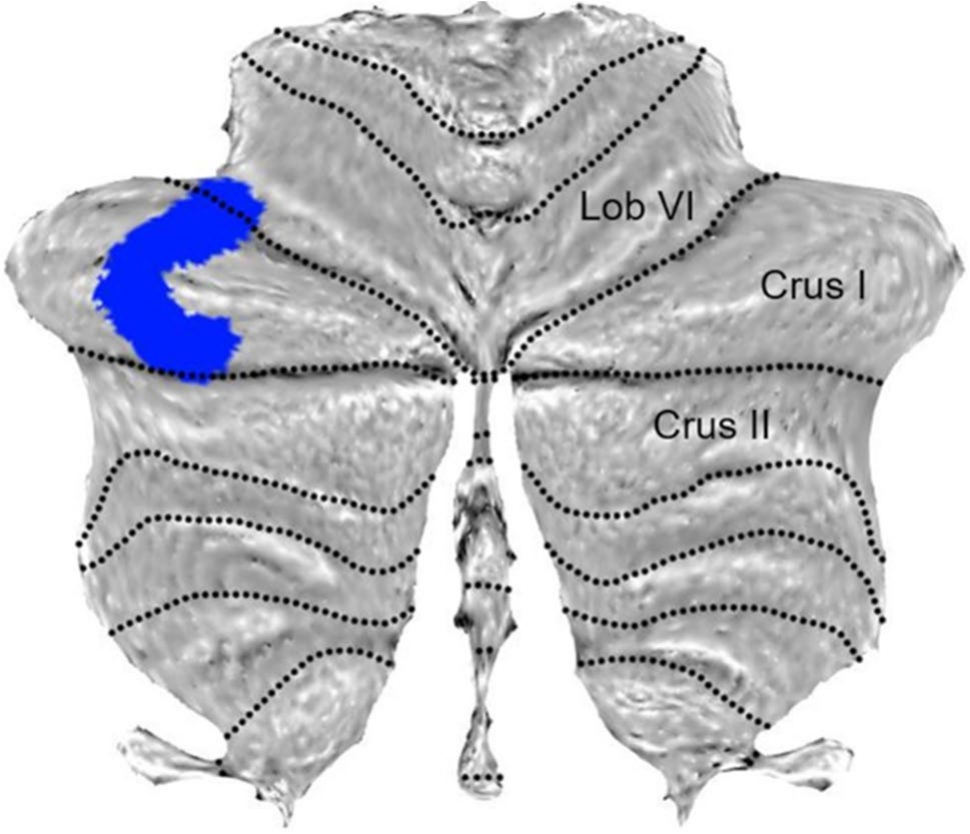
Cerebellar regions showing significant differences in GM volume between groups ([older > young] (*p* < 0.05 FWE corrected for multiple comparisons at cluster level, extended threshold fixed at k = 20 voxels)

**Fig. 2:**
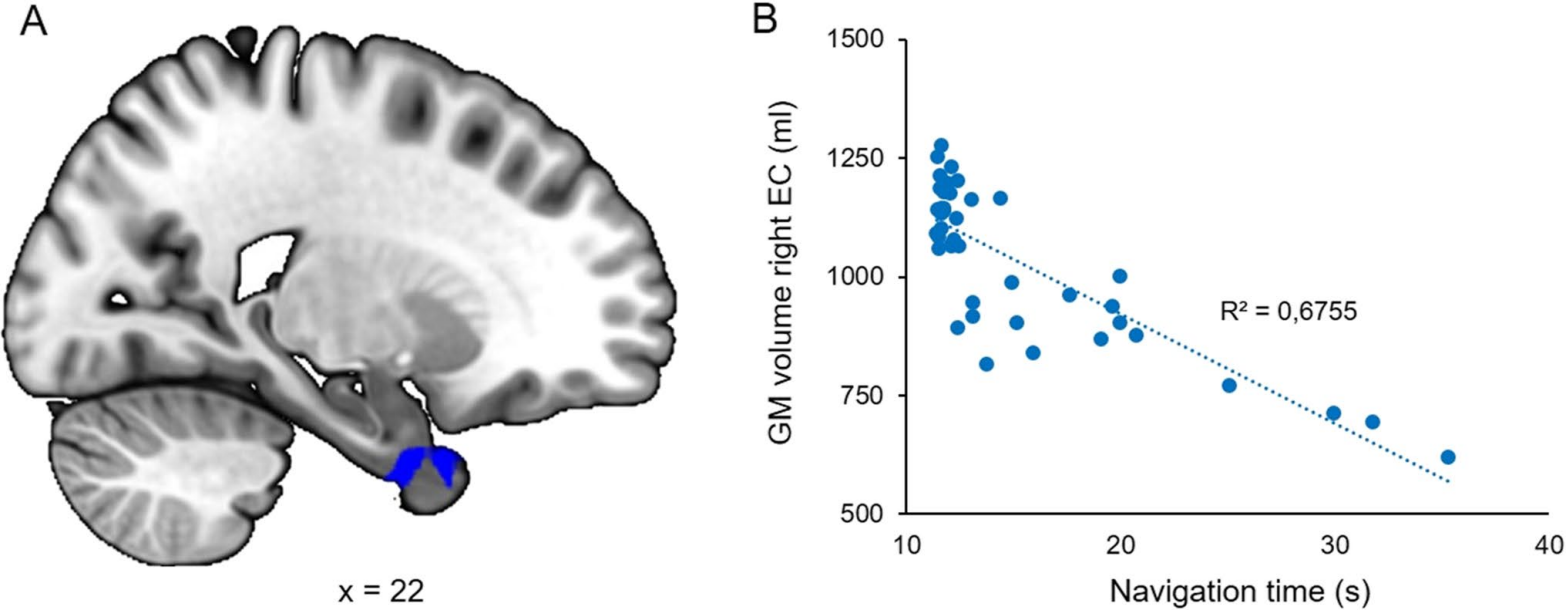
Regression analysis between GM volume and change in navigation time at the whole brain level. **A** Only the anterior part of the right temporal lobe including the entorhinal cortex showed a significant negative association with navigation time. Statistical threshold *p* < 0.05 FWE corrected for multiple comparisons at cluster level, extended threshold fixed at k = 20 voxels. **B** Graphical representation of the association

Of note, the previous fMRI study [12] revealed that older adults were slower to reach the goal, that they made more errors, and that they relied more on response strategies than young adults during navigation. Moreover, older participants had significantly lower scores than young participants across all neuropsychological measures (MMSE, 3D, Corsi, and PPT). Comparison between age groups revealed a single cluster with significantly smaller GM volume (k = 2009 voxels; *p* < 0.001 FWE cluster-level) in the left neocerebellum of healthy older participants (Fig. 1). This cluster contained one peak in Crus I (t = 5.19; peak coordinate xyz: -35 -64 -36) and one peak in the caudal part of lobule VI (t = 4.79; xyz: -38 -58 -27). We then performed correlational analyses between navigation performance and GM volume as well as between neuropsychological scores and GM volume. Results showed no significant association with navigational performance but a negative association between PPT scores and GM volume in lobule VI of the right neocerebellum (t = 4.55; k = 1557; *p* = 0.015 FWE cluster-level; xyz: 11 -63 -19). No other significant association between neuropsychological scores and GM volume was found. A supplementary whole-brain analysis showed that reduced GM volume in the right entorhinal cortex only was related to an increase in navigation time (Fig. 2; t = 4.37; k = 2131; p = 0.002 FWE cluster-level; xyz: 22 2 -36).

## Discussion

Despite growing evidence underlining the importance of the neocerebellum for complex cognitive behavior such as spatial navigation, few studies have sought to investigate how healthy aging could modulate cerebellar GM integrity. Our results showed age-related decrease of GM volume in the Crus I and in the caudal part of lobule VI of the left cerebellum. These regions have been reported to be mainly involved in cognitive processes such as visual object recognition, divided attention, and response selection [4]. The lateral part of Crus I is implicated in three intrinsically connected circuits: the central executive (fronto-parietal), the default mode, and the ventral attentional networks [6]. More specifically, left Crus I plays a part in a subnetwork dedicated to spatial navigation that encompasses the posterior parietal cortex along with the retrosplenial, hippocampal, and entorhinal cortices. It could be involved in place-based navigational strategy, allocentric-to-egocentric translation, and mental navigation, cognitive skills that are essential for adequate spatial navigation (reviewed by [23]). Rodent studies have also highlighted the involvement of Crus I in learning and goal-directed behavior [24]. Left lobule VI overlaps with the attentional cerebellar subnetwork that supports stimulus-driven and goal-directed behavior as well as executive processing in human [7, 25]. In addition, animal studies have found that the stimulation of lobule VI in mice altered spatial memory performance and object-location processing in CA1 [26].

The left Crus I atrophy (xyz: -35 -64 -36) found in older adults could be interpreted in light of previous fMRI results from [12]. Ramanoël and colleagues revealed, with the same participants, that the right Crus I (xyz: 36 -73 -22) was more activated in older adults than young adults during spatial navigation for the fMRI contrast [navigation > fixation]. The within group analysis of the fMRI contrast [navigation > fixation] showed significant cerebellar clusters in the right Crus I overlapping lobule VI (xyz: 33 -43 -28) and in the left lobule IX (xyz: -15 -52 -46) of older adults. The right lateralized activation of Crus I could constitute a functional adaptation to the smaller GM volume in the left Crus I in healthy aging. Igloi and colleagues [11] highlighted that the left cerebellum, including Crus I, and the right hippocampus subtended the use of a place-based strategy, whereas the right Crus I and the left hippocampus were involved in the use of a sequence-based strategy (i.e., a response-based strategy). Smaller GM volume in the left Crus I of older adults may thus provide an explanation for the age-related shift in navigational strategy preference from place to response strategies [12, 27] and more generally to older adults’ reduced spatial navigation ability. It must be noted that we did not assess specific executive functions such as attention or mental flexibility and therefore cannot rule out the possibility that age-related differences in executive abilities contributed to this strategy shift. Future neuroimaging studies should investigate this relationship precisely by considering the activity as well as the morphometry of the neocerebellum.

Giving support to this hypothesis, our results appear to overlap with the functional task-related cerebellar atlas for the use of a basic spatial map by King and colleagues (“Spatial_Map_Easy_MDTB34”). We need to highlight that the present study failed to identify a significant association between navigational performance and GM volume in the cerebellum. These results could be partially explained by the limited sample size but also by the relative simplicity of the navigation paradigm involving a two-choice task based on a small number of simple objects. However, we did show significant association between GM volume and scores on the PPT in lobule VI. This result and the absence of other significant correlations, notably with tests assessing specific memory processes, could be also explained by the capacity of the PPT to reflect multiple facets of cognition [28, 29]. Previous studies have shown that the performance of older adults in navigational tasks could be predicted by PPT scores but not by executive or memory scores [30]. This in line with the supplementary whole-brain analysis showing a negative association between navigation time and GM volume of the right entorhinal cortex, a key region for general navigation abilities. Interestingly, this structure is particularly vulnerable to normal and pathological aging, and it is associated with impaired navigational performance in healthy older adults [31].

These preliminary findings warrant further research with a larger sample size and ecological navigation paradigms that could disentangle the cognitive and motor aspects of spatial cognition. Despite several limitations, our results reveal age-related atrophy in Crus I and Lobule VI, regions which are specifically implicated in the cognitive aspects of spatial navigation [23]. We emphasize the importance of considering cerebellar regions to better comprehend changes in spatial navigation ability in normal and pathological aging [32].
